# Explosive Medicines

**Published:** 1874-12

**Authors:** 


Explosive Medicines.—Young medical men are warned (*Revue de Therapie,* 1873,) against combining in their prescriptions certain agents which, in case of the development of any acid, or even if exposed to a moderately high temperature, are liable to *go off.* Among these dangerous formulæ may be cited a prescription for pills not unfrequently employed in England, composed of nitrate of silver, extract of nux vomica, muriate of morphia, conserve of roses and extract of gentian, which, when affected by the development of heat, will speedily explode. In like manner, pills made of nitrate of silver and creasote, or carbolic acid, will very soon generate heat sufficient to produce spontaneous combustion. Still more surprising to the occupants of the sick chamber is the energetic explosion (suggestive of nitro-glycerine) arising from the pills or mixtures of which oxymuriate of potash forms an ingredient.

